# Supplementary material for: Regulation of PCNA cycling on replicating DNA by RFC and RFC-like complexes
Source: Nat Commun. 2019 Jun 3;10:2420. doi: 10.1038/s41467-019-10376-w (PMC6546911; doi:10.1038/s41467-019-10376-w)
Supplement: Supplementary file 1 — Supplementary information [file 41467_2019_10376_MOESM1_ESM.pdf]

Supplementary Information

Regulation of PCNA cycling on replicating DNA by RFC and RFC-like complexes

Kang et al.

Supplementary Figures

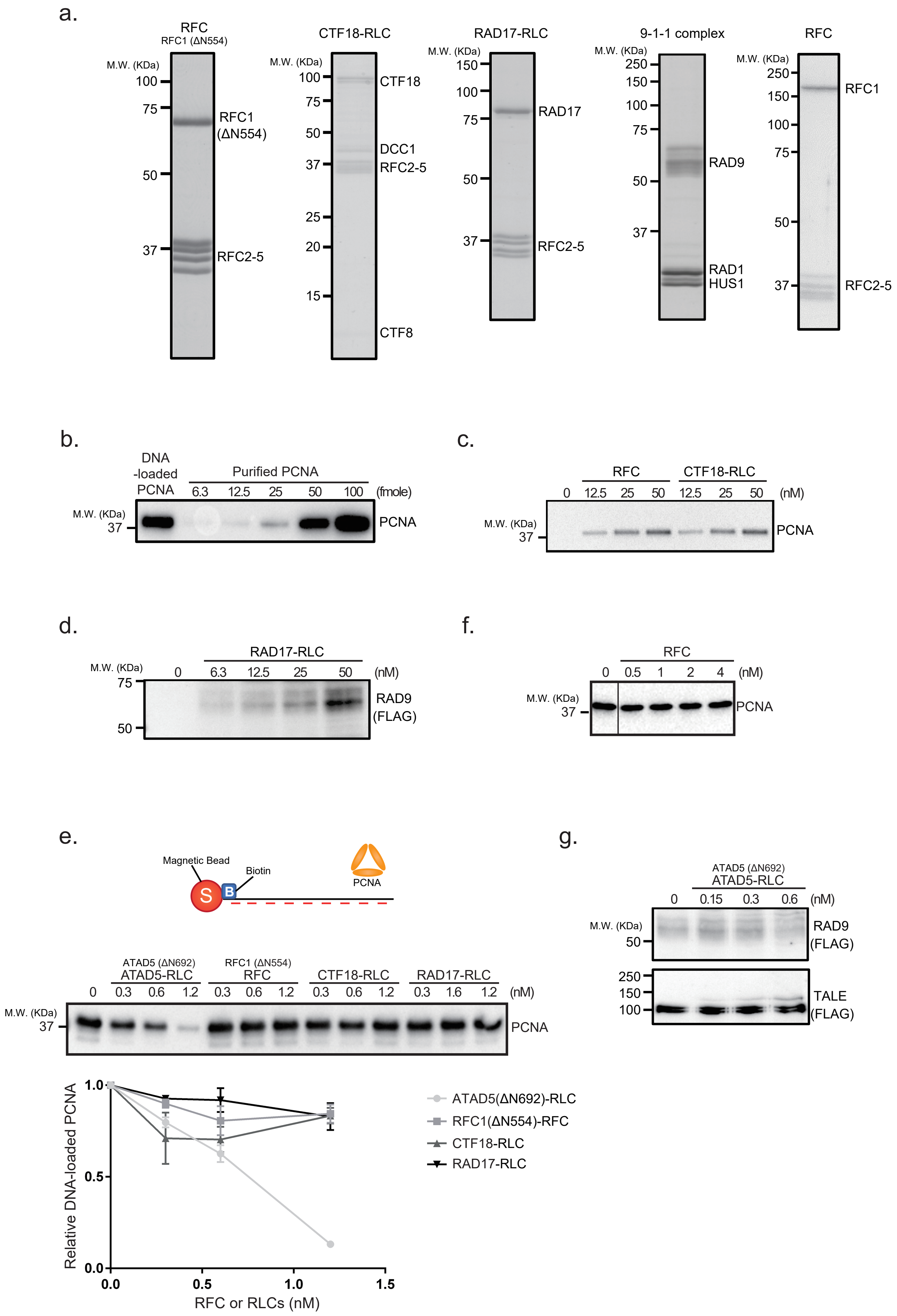

Supplementary Figure 1.

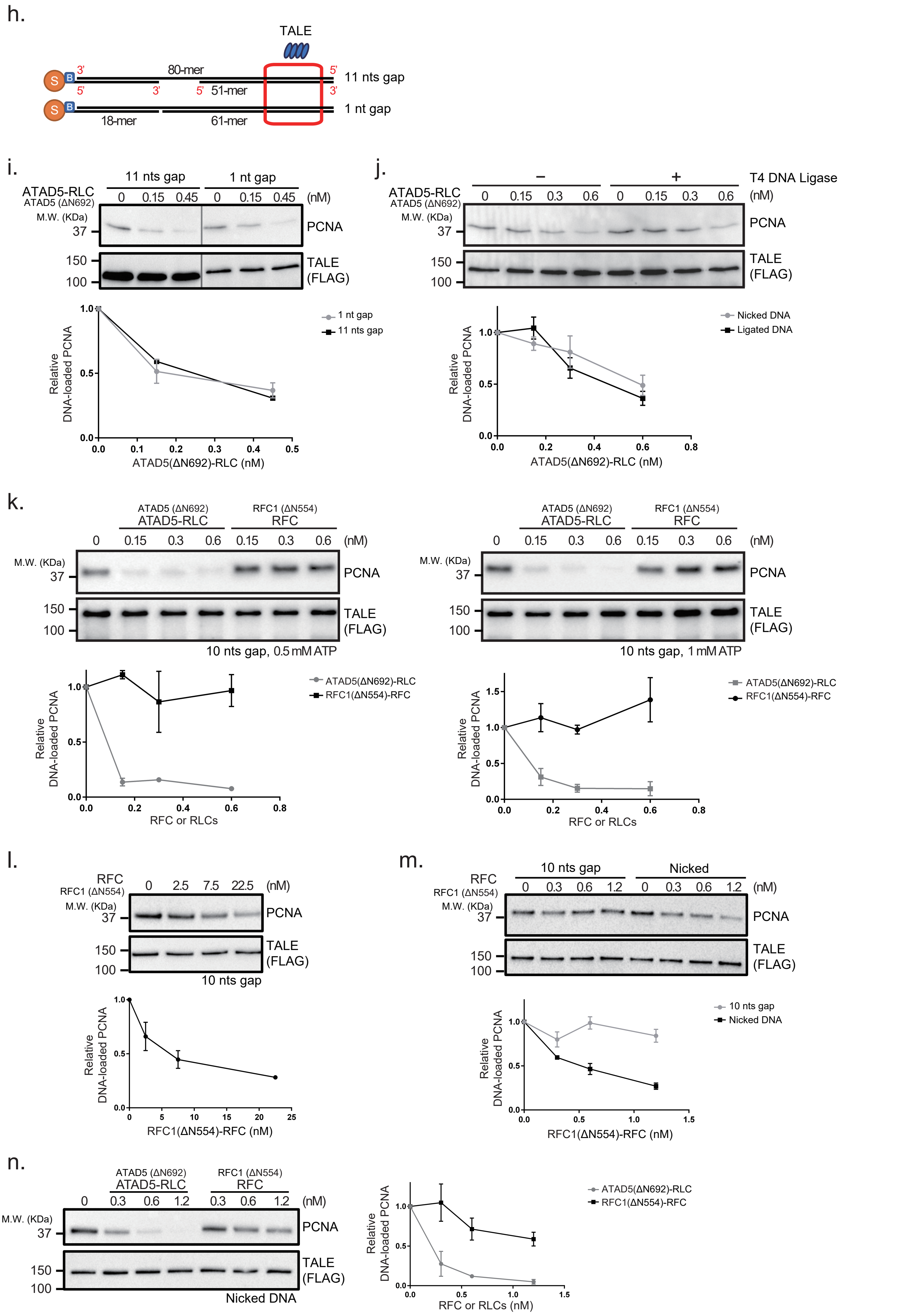

Supplementary Figure 1.

Supplementary Figure 1. Biochemical activities of RFC and RFC-like complexes.

a. Coomassie-stained SDS-PAGE of purified RFC, RFC1 (ΔN554)-RFC, CTF18-RLC, RAD17-RLC and RAD9-HUS1-RAD1. **b.** PCNA loading by RFC1 (ΔN554)-RFC. DNA-loaded PCNA was quantified after immunoblotting. **c.** CTF18-RLC loads PCNA to primer-template DNA. Indicated amounts of RFC (full-length RFC1) and CTF18-RLC were examined in the PCNA loading assay. **d.** Loading of RAD9-HUS1-RAD1 (human 9-1-1 complex) by RAD17-RLC. Purified RAD9-HUS1-RAD1 complexes were loaded to the primer-template DNA by indicated amount of RAD17-RLC. Loading of human 9-1-1 complexes were analyzed by immunoblotting for FLAG-RAD9. **e.** ATAD5-RLC unloads PCNA. PCNA-unloading assay was performed with RFC and RLCs. 1.4 Kbps DNA was used for this assay. Graph shows relative PCNA amounts remained on DNA after unloading reaction (n=2). **f.** PCNA unloading reaction was performed with full-length RFC. **g.** ATAD5-RLC does not unload human 9-1-1 complex. The indicated amounts of ATAD5-RLC were treated to the DNA-loaded human 9-1-1 complex. **h-j.** DNA structure did not affect PCNA unloading by ATAD5-RLC. **(h)** DNA substrates used for PCNA-unloading assay in Supplementary Fig. 1i. Each 80-mer DNA substrate has a 1 or 11 nucleotides gap, respectively. The biotinylated side of DNA was attached to the streptavidin-coated magnetic bead. TALE-binding sites were introduced to the other side to prevent the loaded PCNA from sliding off. Substrate DNAs were pre-incubated with MBP-FLAG-TALE before the PCNA-loading and unloading reaction. **(i)** DNA gap-size does not affect PCNA unloading by ATAD5-RLC. PCNA unloading from DNA substrates shown in Supplementary Fig. 1h were examined. Similar amount of the DNA-loaded PCNA for each substrate was used for the unloading assay. Graph shows relative PCNA amounts remained on DNA after unloading reaction (n=2). **(j)** ATAD5-RLC unload PCNA from nicked DNA and duplexed DNA with similar efficiency. PCNA was loaded to 130-mer DNA containing a nick. After loading, PCNA-loaded DNA was incubated with T4 DNA ligase to seal the nick. After ligation reaction, PCNA-unloading reaction was performed. Graph shows relative PCNA amounts remained on DNA after unloading reaction (n=3). **k.** Effect of ATP concentration on PCNA unloading. 0.5 mM or 1 mM ATP was used in PCNA unloading reaction. Reaction was performed with 10-nucleotide-gap DNA (130-mer). ATAD5-RLC unloaded PCNA efficiently at low ATP concentrations, but RFC did not. Graph shows relative PCNA amounts remained on DNA after unloading reaction (n=2). **l.** RFC unloads PCNA from gapped DNA at high concentrations. Reaction was performed with 10-nucleotide-gap DNA (130-mer) and 1 mM ATP. Graph shows relative PCNA amounts remained on DNA after unloading reaction (n=2). **m.** RFC unloads PCNA more efficiently from nicked DNA compared 10-nucleotide-gap DNA. **n.** PCNA-unloading activity of RFC is significantly lower than that of ATAD5-RLC. Reaction

was performed with indicated 130-mer DNA substrate and 1 mM ATP. Graph shows relative PCNA amounts remained on DNA after unloading reaction (n=2).

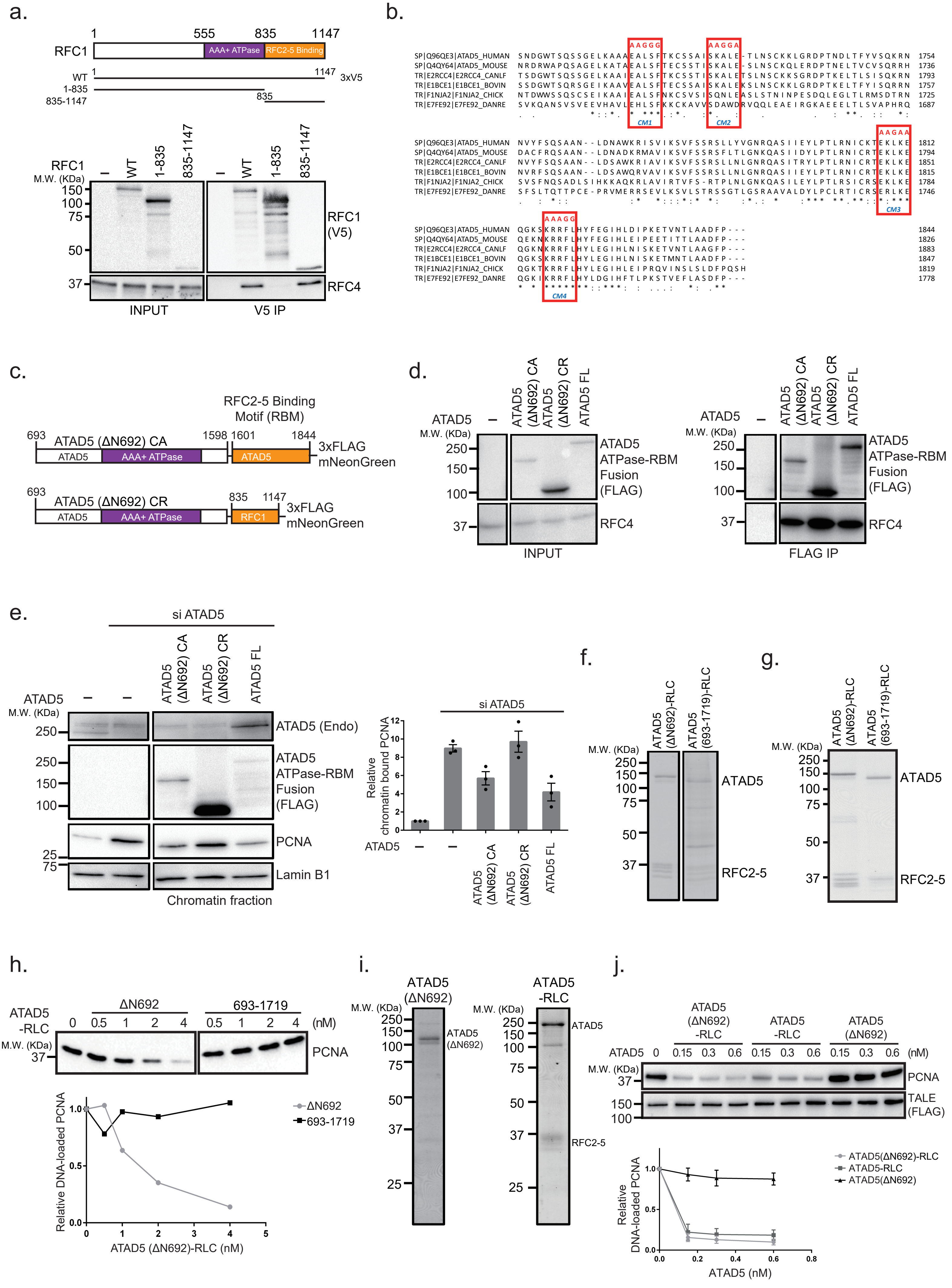

Supplementary Figure 2.

Supplementary Figure 2. Proper binding of RFC2–5 to ATAD5 is necessary for the formation of unloading-competent ATAD5-RLC.

a. The C-terminal domain of RFC is important for RFC2–5 binding. The 3×V5-tagged RFC1 variants were transiently transfected to human 293T cells. After V5-immuno-precipitation, RFC2–5 binding was monitored by co-immunoprecipitation of RFC4. RFC1 (835–1147) was sufficient for RFC2–5 binding. **b.** Locations of CM mutants in RBM of ATAD5. Four five-amino-acid stretches in RBM, which are conserved among species, were mutated as indicated. **c.** Diagram showing RBM-swap mutant of ATAD5. Amino acid numbers are denoted in the diagram. In ATAD5 (ΔN692) CA, a NheI restriction site was introduced at residues 1599–1600 of ATAD5 (ΔN692). RBM of RFC1 was fused to ATAD5 (693-1598) after the introduced NheI site in ATAD5 (ΔN692) CR. **d.** RBM-swap mutant of ATAD5 binds to RFC2–5. RFC2–5 binding to the indicated ATAD5 variants were examined by co-immunoprecipitation of RFC4. **e.** RBM-swap mutant of ATAD5 is defective in PCNA unloading. ATAD5 (ΔN692) CA or CR was expressed in ATAD5-depleted cells, and the amount of chromatin-bound PCNA was analyzed. Graph indicates relative PCNA amount on the chromatin (n=3). **f.** Coomassie-stained SDS-PAGE of the purified RBM-deletion mutant. ATAD5 (ΔN692) or ATAD5 (693–1719) was co-expressed with RFC2–5 in insect cells for purification. **g.** Coomassie-stained SDS-PAGE of ATAD5 (ΔN692) and ATAD5 (693–1719) purified from the combination of yeast and bacterial expression system in the presence of RFC2-5. **h.** RBM of ATAD5 is crucial for the formation of unloading-competent ATAD5-RLC. PCNA unloading assay was performed with ATAD5 (ΔN692)-RLC and ATAD5 (693–1719) purified from the yeast-bacterial expression system. Graph shows relative PCNA amounts remained on DNA after unloading reaction. **i.** ATAD5 (ΔN692) alone and full-length ATAD5-RLC purified from the yeast-bacterial expression system. **j.** PCNA unloading assay was performed with ATAD5 (ΔN692)-RLC, full-length ATAD5-RLC, and ATAD5 (ΔN692) alone that are purified from the yeast-bacterial expression system. Graph shows relative PCNA amounts remained on DNA after unloading reaction (n=3). ATAD5 alone did not unload PCNA. Full length ATAD5-RLC and ATAD5 (ΔN692)-RLC unloaded PCNA with similar efficiency.

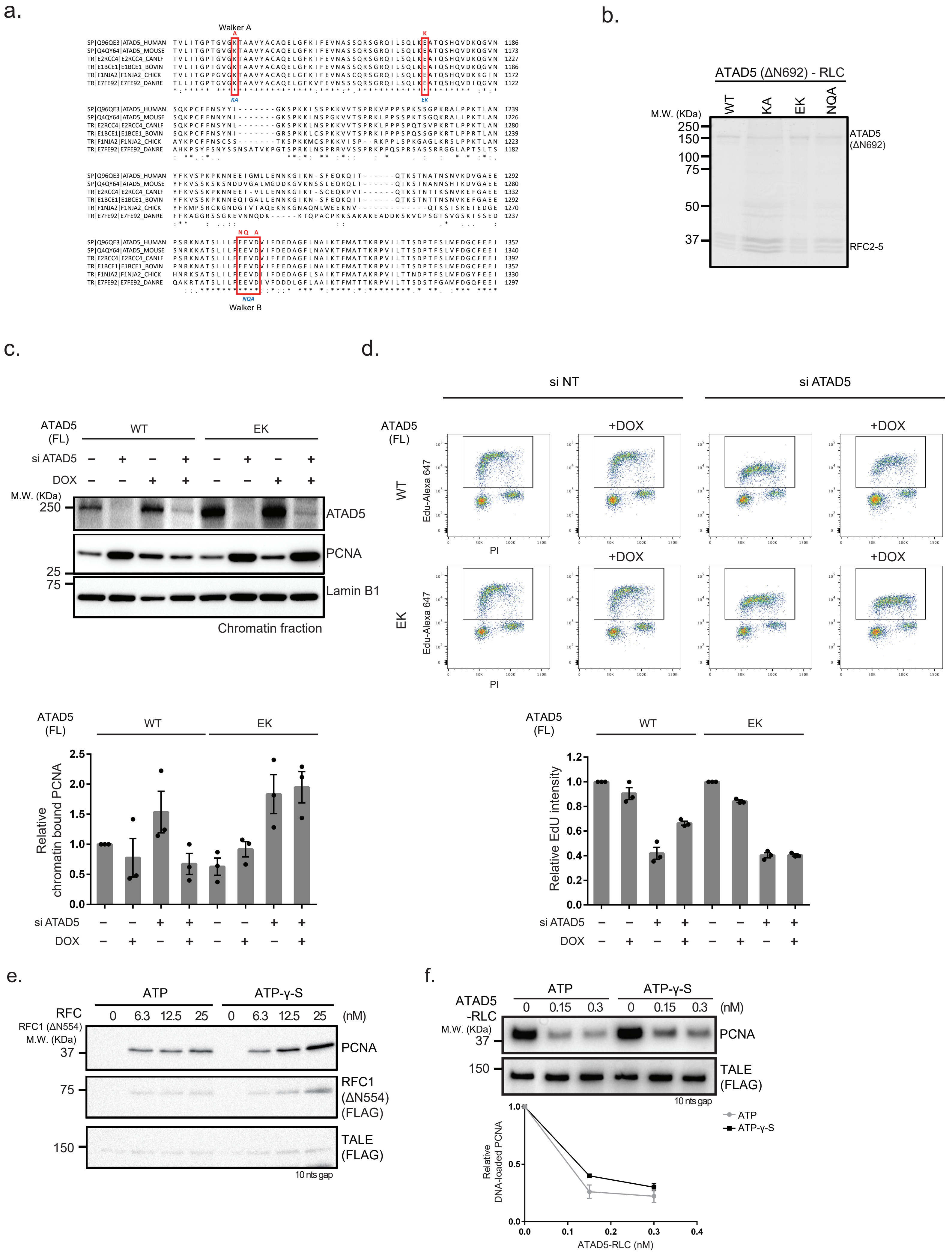

Supplementary Figure 3.

Supplementary Figure 3. ATP binding and hydrolysis control PCNA loading and unloading reaction.

a. Positions of KA, NQA, and EK mutations in human ATAD5. Amino acids around Walker A and Walker B motifs of ATAD5 were well-conserved among species. **b.** Coomassie-stained SDS-PAGE gel of purified wild-type ATAD5 (ΔN692)-RLC and ATPase-motif mutants. **c-d.** PCNA unloading is important for S phase progression. Doxycycline-inducible wild type or EK mutant of ATAD5 was integrated in U2OS cells using lentiviral vector. After doxycycline induction and endogenous ATAD5 depletion, cells were pulse-labeled with EdU to monitor DNA replication. (**c**) EK mutant failed to unload PCNA. Chromatin bound PCNA and ATAD5 were analyzed by immunoblot after chromatin fractionation. Graph indicates relative PCNA amount on the chromatin (n=3). (**d**) EdU incorporation by cells expressing wild type ATAD5 or EK mutant were analyzed by flow cytometry. Graph shows quantification of EdU intensity (n=3). ATAD5-depletion caused a significant reduction of EdU. Unloading-defective EK mutant could not restore EdU incorporation compared to wild-type ATAD5. **e.** ATP-γ-S stalls PCNA-loading reaction at the initial binding of RFC-PCNA to DNA. PCNA-loading reaction was performed with 10-nucleotide-gap DNA (130-mer) in the presence of ATP or ATP-γ-S. RFC and PCNA were both accumulated on substrate DNA with ATP-γ-S. **f.** Comparison of PCNA unloading activity of full-length ATAD5-RLC in presence of ATP or ATP-γ-S. Graph shows relative PCNA amounts remained on DNA after unloading reaction (n=2).

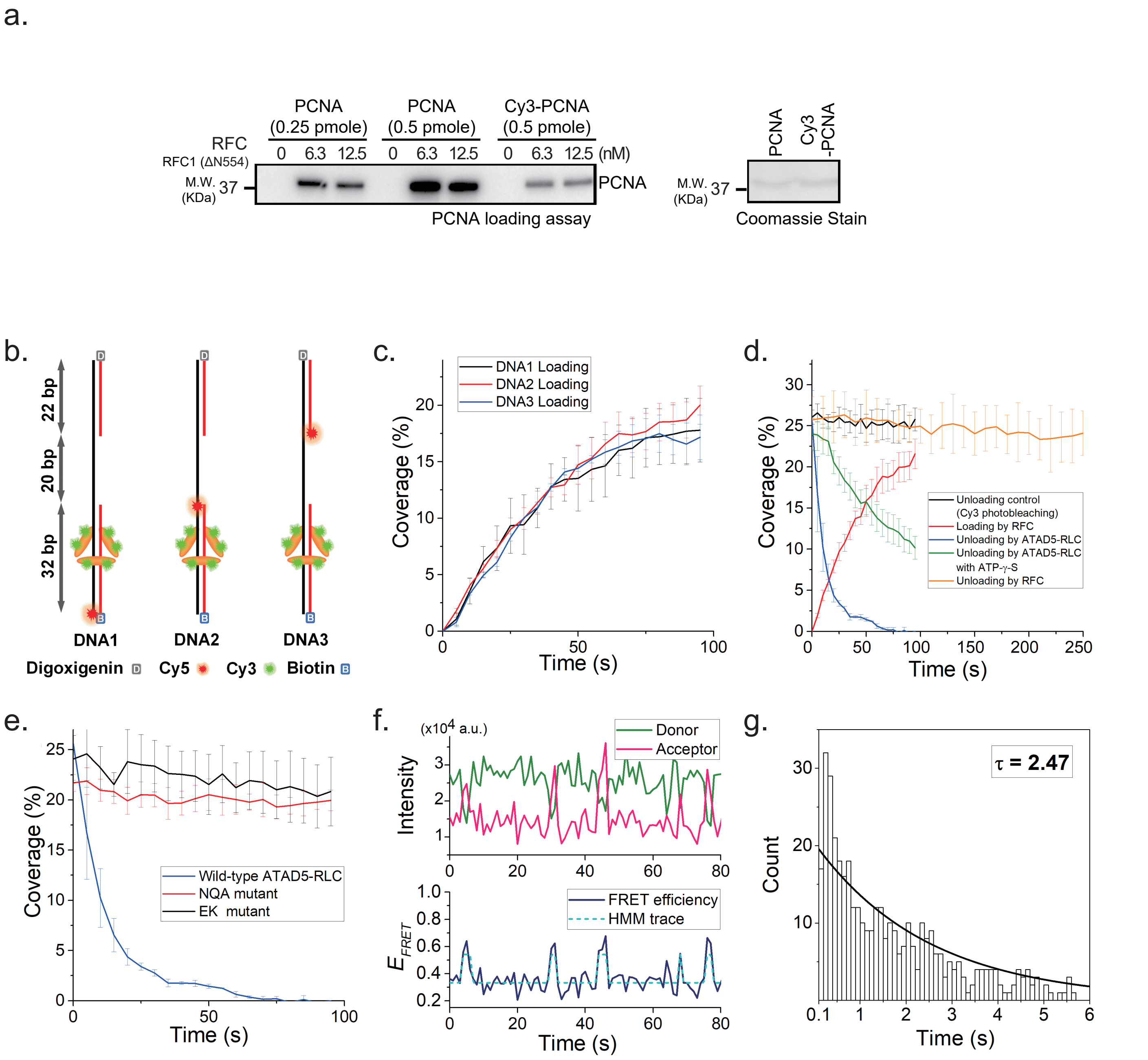

Supplementary Figure 4.

h.

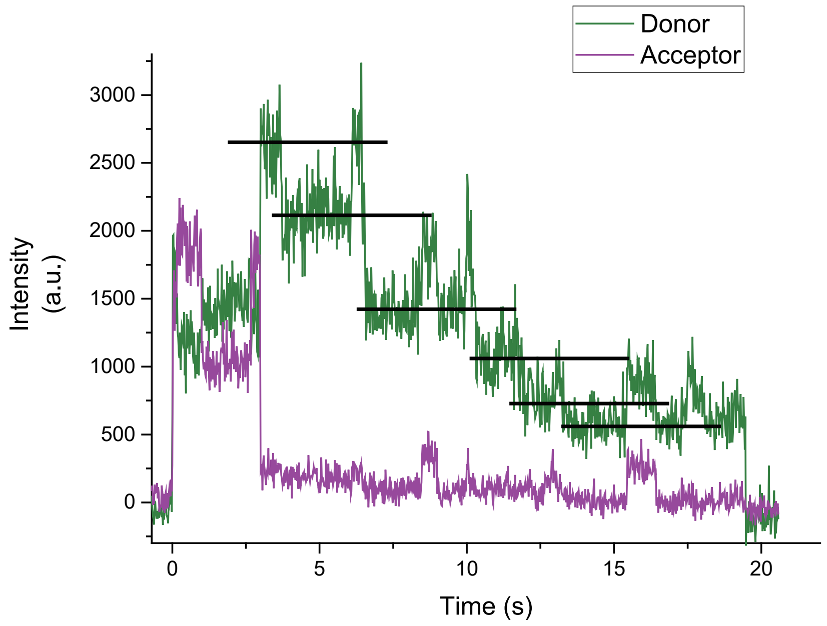

i.

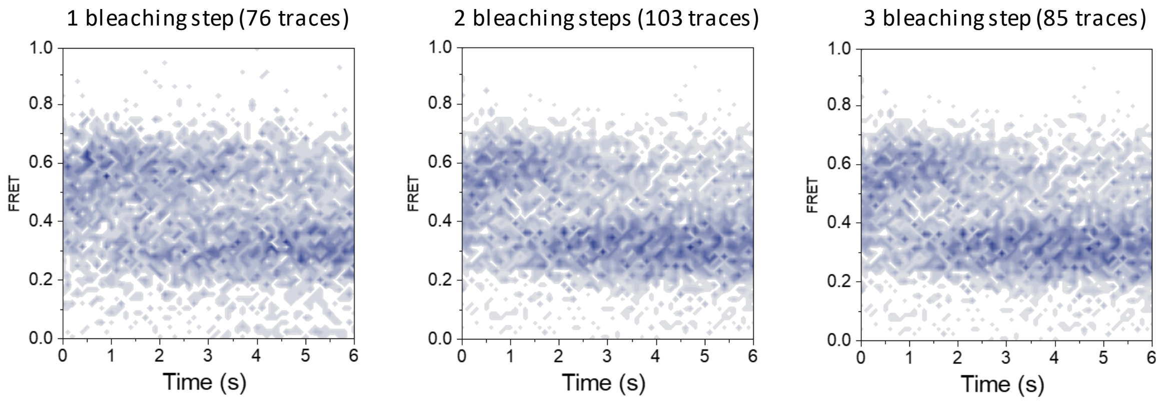

j.

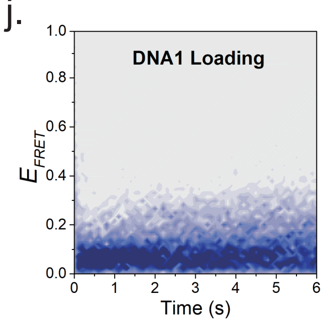

k.

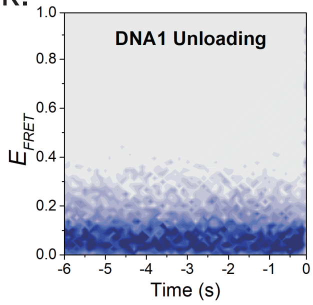

l.

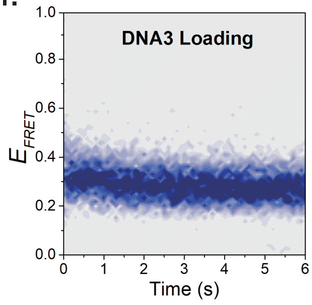

m.

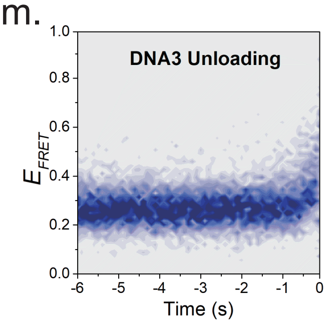

Supplementary Figure 4.

Supplementary Figure 4. Molecular dynamics of PCNA-unloading by ATAD5-RLC.

a. *Left panel:* Ensemble PCNA loading reaction using Cy3-labled PCNA. Indicated amount of unlabeled PCNA or Cy3-labeled PCNA were examined in PCNA loading reaction. *Right panel:* Unlabeled PCNA and Cy3-labeled PCNA were analyzed by SDS PAGE followed by Coomassie staining. **b.** Schematic diagram showing the template designs with varying Cy5 positions. DNA2 was used unless otherwise noted. **c.** Growth of PCNA coverage since flowing in pre-assembled RFC-PCNA complex. Coverage indicates the percentage of DNA spots that were co-localized with PCNA spots. Error bars represent s.e.m. from triplicate measurements. All three DNA templates exhibited similar loading curves, suggesting that Cy5 position on DNA does not to affect the loading dynamics. **d.** Change of PCNA coverage from loading with RFC (red; same as loading on DNA2 in (**c**)), unloading by ATAD5-RLC (blue; flowing in ATAD5-RLC to pre-loaded PCNA), unloading by ATAD5-RLC with ATP-γ-S (green), unloading by RFC (orange; flowing in RFC to pre-loaded PCNA), and unloading control (black; unloading without any protein complex) which shows minimal decay due to Cy3 photobleaching. **e.** Unloading curve for NQA and EK mutants of ATAD5, compared to wild type, shows no unloading activity. **f.** Representative smFRET trace for pre-loaded PCNA on DNA2, showing mostly a stable FRET level at 0.60 with transient spikes. **g.** Dwell time distribution of the transient events of FRET spikes collected from 88 traces. **h.** An example FRET trace showing the loading of a PCNA trimer that contains 6 Cy3 dyes, represented by 6 photobleaching steps. Cy5 (acceptor) photobleaching was followed by 6 consecutive photobleaching events of Cy3. Black lines indicate the fluorescence levels of each step. **i.** In order to test how the number of donor dyes in a PCNA trimer affects the apparent FRET efficiency, FRET evolution maps were constructed from different groups of FRET traces containing 1, 2, or 3 Cy3 molecules, which were distinguished by the number of photobleaching steps. FRET evolution maps from traces with fewer Cy3 molecules show broader pattern of the FRET population density but the FRET levels of the dominating populations at early and late stages remain consistent, coinciding with the high FRET level (0.62) of LI2 and the low FRET level (0.34) of LS, respectively. The broadening of the population stems from the lower S/N and the variant position of Cy3 dye on the PCNA trimer. The maps from traces with 2 and 3 Cy3 dyes better reveal the short-lived initial mid-FRET population, similar to the map in Figure 4d. **j-k.** Synchronized heat map of 261 loading traces (**j**) and 199 unloading traces (**k**) on DNA1, representing the majority of traces staying at low FRET state. **l-m.** Synchronized heat map of 199 loading traces (**l**) and 190 unloading traces (**m**) on DNA3.

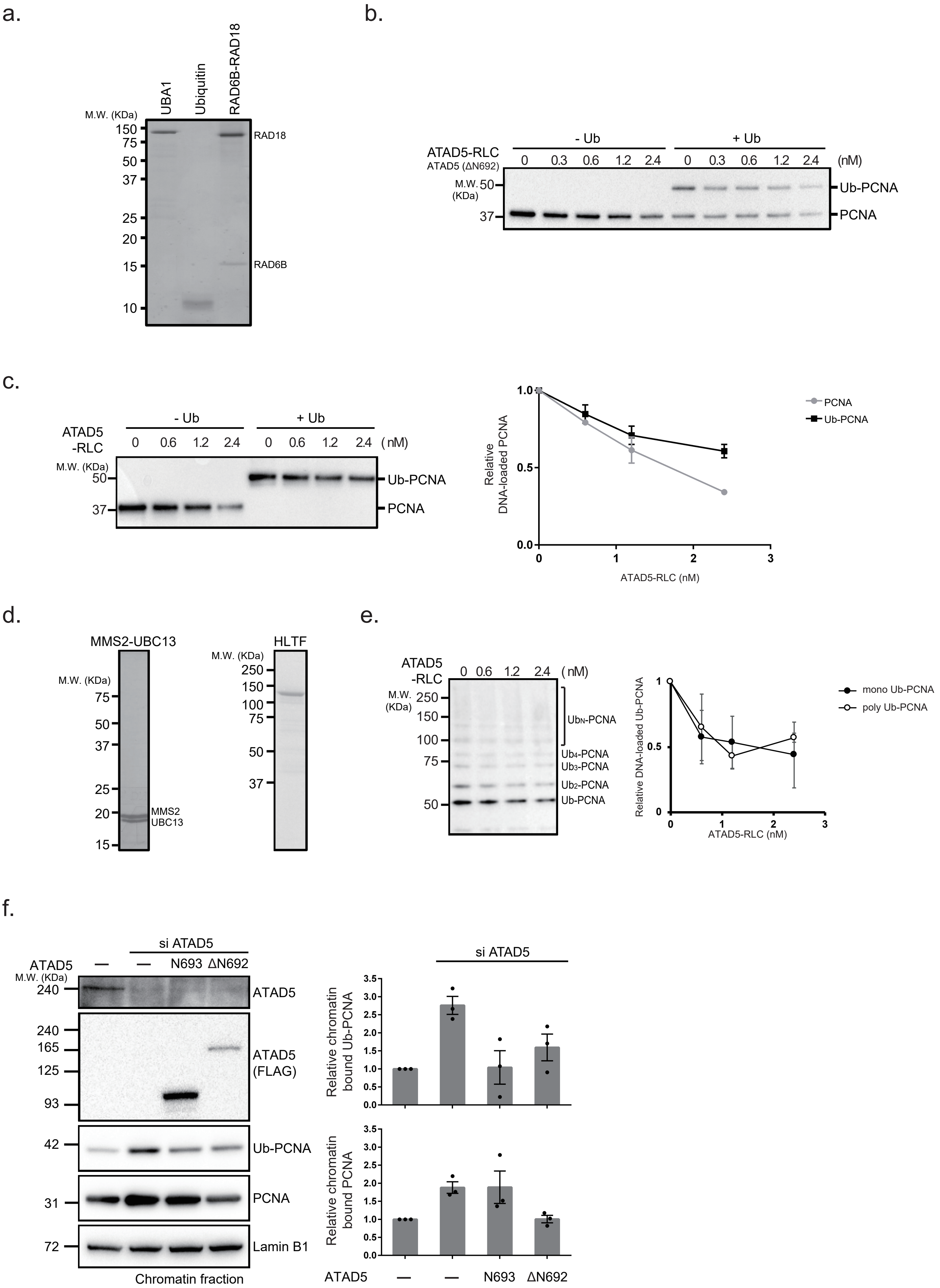

Supplementary Figure 5.

Supplementary Figure 5. Unloading of ubiquitinated PCNA by ATAD5-RLC.

a. Coomassie-stained SDS-PAGE gel for purified UBA1, Ubiquitin and RAD6B-RAD18. **b.** ATAD5 (ΔN692)-RLC unloads PCNA and mono-ubiquitinated PCNA with similar efficiency. The PCNA unloading reaction was performed with partially ubiquitinated PCNA. **c.** Unloading of PCNA or mono-Ub-PCNA by full-length ATAD5-RLC. PCNA-unloading reaction was performed with full-length ATAD5-RLC after PCNA mono-ubiquitination reaction. 1.4 Kbps DNA was used for this assay. Graph shows relative PCNA or mono-Ub-PCNA amounts remained on DNA after unloading reaction (n=2). **d.** Coomassie-stained SDS-PAGE gels of purified MMS2-UBC13 and HLTF. **e.** Unloading of poly-Ub-PCNA by full-length ATAD5-RLC. PCNA-unloading reaction was performed with full-length ATAD5-RLC after PCNA poly-ubiquitination reaction. 10-nucleotide-gap DNA (130-mer) was used for this assay. Graph shows relative amounts of mono-Ub-PCNA or poly-Ub-PCNA remaining on DNA after unloading reaction (n=3). **f.** ATAD5 (ΔN692) reduced chromatin bound mono-ubiquitinated PCNA. N-terminal FLAG-tagged 1st ~ 693rd amino-acid region of ATAD5 (ATAD5 (N693)) or 693rd ~ 1844th amino-acid region of ATAD5 (ATAD5 (ΔN692)) was transiently expressed in ATAD5-depleted cells. Cells were treated with 254 nm UV (50 J/m^2) 6 hours before harvest. Chromatin bound PCNA and mono-ubiquitinated PCNA (Ub-PCNA) were analyzed using anti-PCNA or anti-Ub-PCNA antibody, respectively. ATAD5 (N693) contains a UAF1-binding motif that was crucial for de-ubiquitination of Ub-PCNA. Graph shows relative amount of PCNA or Ub-PCNA on the chromatin (n=3).

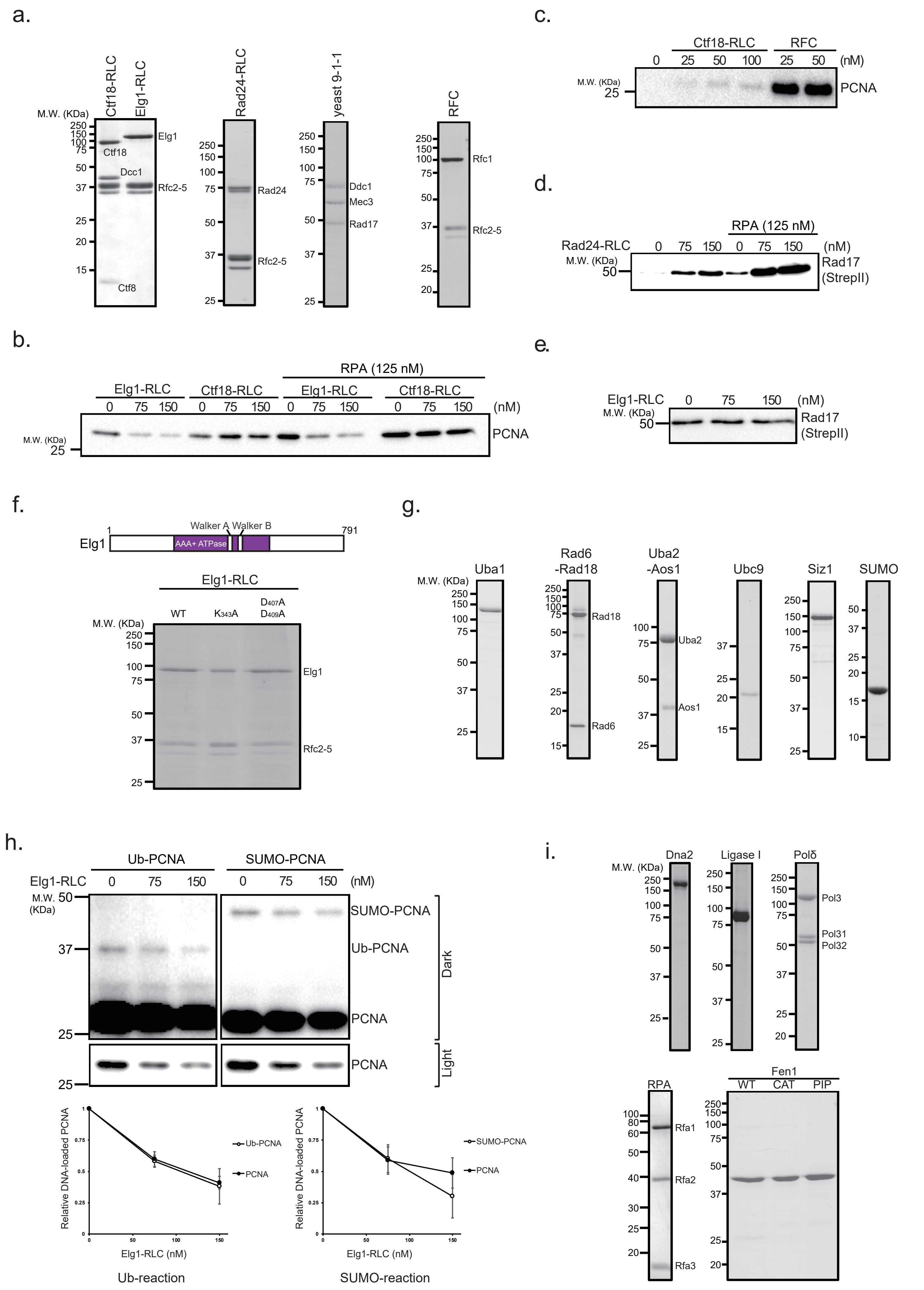

Supplementary Figure 6.

Supplementary Figure 6. Elg1-RLC is an unloader of which function might be regulated by Okazaki fragment-processing enzymes.

a. Coomassie-stained SDS-PAGE gels of purified yeast Ctf18-RLC, Elg1-RLC, Rad24-RLC, Ddc1-Mec3-Rad17, and RFC. **b.** Elg1-RLC unloads PCNA, but Ctf18-RLC does not. PCNA unloading activities of Elg1-RLC or Ctf18-RLC were examined in the presence or absence of RPA. Indicated amount of proteins were added to the PCNA unloading reaction. **c.** Yeast Ctf18-RLC possesses weak PCNA-loading activity. The PCNA loading reaction was performed with the indicated amount of RFC or Ctf18-RLC. **d.** Yeast Rad24-RLC loads Ddc1-Mec3-Rad17. The loading reaction was performed with 1.4 Kbps DNA. Loading of Ddc1-Mec3-Rad17 was monitored by DNA-association of Rad17-Strep II. RPA stabilized the DNA binding of Ddc1-Mec3-Rad17. **e.** Elg1-RLC does not unload DNA-bound Ddc1-Mec3-Rad17. After DNA-loading reaction of Ddc1-Mec3-Rad17, Elg1-RLC was treated to DNA-bound Ddc1-Mec3-Rad17. **f.** Coomassie-stained SDS-PAGE gel of purified wild-type or ATPase-motif-mutant Elg1-RLC. Indicated Elg1 variants were co-expressed with Rfc2–5 in yeast cells and purified through sequential application of anti-FLAG agarose resin and SP Sepharose. **g.** Coomassie-stained SDS-PAGE gels of purified Uba1, Rad6-Rad18, Uba2-Aos1, Ubc9, Siz1, and SUMO. **h.** Elg1-RLC unloads ubiquitinated PCNA and SUMOylated PCNA. PCNA was either ubiquitinated (Ub-PCNA) or SUMOylated (SUMO-PCNA) with purified enzymes. DNA-loaded Ub- and SUMO-PCNA were treated with Elg1-RLC. Graph shows relative PCNA, Ub-PCNA or SUMO-PCNA amounts remained on DNA after unloading reaction (n=3). Ub- and SUMO-PCNA were unloaded with a similar efficiency compared with unmodified PCNA. **i.** Coomassie-stained SDS-PAGE gels of purified Dna2, Ligase I, Polymerase δ, RPA and wild-type or mutant Fen1.

Supplementary Tables

Supplementary Table 1. Yeast strains used in this study

Strain	*Genotype*	Source
yMBS001	*ade2-1 trp1-1 leu2-3,112 his3-11,15 ura3-1 can1-100 bar1::HisG lys2::HisG pep4Δ::unmarked his3::pYMBS001 (GAL1,10-RFC2, RFC3) ura3::pYMBS002 (GAL1,10-RFC4, RFC5) leu2::pYMBS003 (GAL1,10-RFC1-3XFLAG)*	This Study
yMBS002	*ade2-1 trp1-1 leu2-3,112 his3-11,15 ura3-1 can1-100 bar1::HisG lys2::HisG pep4Δ::unmarked his3::pYMBS001 (GAL1,10-RFC2, RFC3) ura3::pYMBS002 (GAL1,10-RFC4, RFC5) leu2::pYMBS004 (GAL1,10-CTF18-3XFLAG) trp1::pYMBS005 (GAL1,10-DCC1, CTF8)*	This Study
yMBS003	*ade2-1 trp1-1 leu2-3,112 his3-11,15 ura3-1 can1-100 bar1::HisG lys2::HisG pep4Δ::unmarked his3::pYMBS001 (GAL1,10-RFC2, RFC3) ura3::pYMBS002 (GAL1,10-RFC4, RFC5) leu2::pYMBS006 (GAL1,10-RAD24-3XFLAG)*	This Study
yMBS005	*ade2-1 trp1-1 leu2-3,112 his3-11,15 ura3-1 can1-100 bar1::HisG lys2::HisG pep4Δ::unmarked his3::pYMBS001 (GAL1,10-RFC2, RFC3) ura3::pYMBS002 (GAL1,10-RFC4, RFC5) leu2::pYMBS007 (GAL1,10-ELG1-3XFLAG)*	This Study
yMBS006	*ade2-1 trp1-1 leu2-3,112 his3-11,15 ura3-1 can1-100 bar1::HisG lys2::HisG pep4Δ::unmarked his3::pYMBS001 (GAL1,10-RFC2, RFC3) ura3::pYMBS002 (GAL1,10-RFC4, RFC5) leu2::pYMBS008 (GAL1,10-ELG1[K343A]-3XFLAG)*	This Study
yMBS009	*ade2-1 trp1-1 leu2-3,112 his3-11,15 ura3-1 can1-100 bar1::HisG lys2::HisG pep4Δ::unmarked his3::pYMBS001 (GAL1,10-RFC2, RFC3) ura3::pYMBS002 (GAL1,10-RFC4, RFC5) leu2::pYMBS011 (GAL1,10-ELG1[D407A, D409A]-3XFLAG)*	This Study
yMBS012	*ade2-1 trp1-1 leu2-3,112 his3-11,15 ura3-1 can1-100 bar1::HisG lys2::HisG pep4Δ::unmarked his3::pYMBS0014 (GAL1,10-MEC3) ura3::pYMBS015 (GAL1,10-RAD17-2XSTREPII) leu2::pYMBS016 (GAL1,10-3XFLAG-DDC1)*	This Study
yMBS013	*ade2-1 trp1-1 leu2-3,112 his3-11,15 ura3-1 can1-100 bar1::HisG lys2::HisG pep4Δ::unmarked leu2::pYMBS017 (GAL1,10-UBA1-3XFLAG)*	This Study
yMBS014	*ade2-1 trp1-1 leu2-3,112 his3-11,15 ura3-1 can1-100 bar1::HisG lys2::HisG pep4Δ::unmarked ura3::pYMBS018 (GAL1,10-RAD6) leu2::pYMBS019 (GAL1,10-RAD18-3XFLAG)*	This Study

yMBS015	*ade2-1 trp1-1 leu2-3,112 his3-11,15 ura3-1 can1-100 bar1::HisG lys2::HisG pep4Δ::unmarked ura3::pYMBS020 (GAL1,10-AOS1) leu2::pYMBS021 (GAL1,10-UBA2-3XFLAG)*	This Study
yMBS016	*ade2-1 trp1-1 leu2-3,112 his3-11,15 ura3-1 can1-100 bar1::HisG lys2::HisG pep4Δ::unmarked leu2::pYMBS022 (GAL1,10-UBC9-3XFLAG)*	This Study
yMBS017	*ade2-1 trp1-1 leu2-3,112 his3-11,15 ura3-1 can1-100 bar1::HisG lys2::HisG pep4Δ::unmarked leu2::pYMBS023 (GAL1,10-SIZ1-3XFLAG)*	This Study
yMBS018	*ade2-1 trp1-1 leu2-3,112 his3-11,15 ura3-1 can1-100 bar1::HisG lys2::HisG pep4Δ::unmarked leu2::pYMBS024 (GAL1,10-DNA2-3XFLAG)*	This Study
yMBS019	*ade2-1 trp1-1 leu2-3,112 his3-11,15 ura3-1 can1-100 bar1::HisG lys2::HisG pep4Δ::unmarked leu2::pYMBS025 (GAL1,10-CDC9-3XFLAG)*	This Study
yMBS020	*ade2-1 trp1-1 leu2-3,112 his3-11,15 ura3-1 can1-100 bar1::HisG lys2::HisG pep4Δ::unmarked leu2::pYMBS025 (GAL1,10-FEN1-3XFLAG)*	This Study
yMBS021	*ade2-1 trp1-1 leu2-3,112 his3-11,15 ura3-1 can1-100 bar1::HisG lys2::HisG pep4Δ::unmarked leu2::pYMBS025 (GAL1,10-FEN1[D179A]-3XFLAG)*	This Study
yMBS022	*ade2-1 trp1-1 leu2-3,112 his3-11,15 ura3-1 can1-100 bar1::HisG lys2::HisG pep4Δ::unmarked leu2::pYMBS025 (GAL1,10-FEN1[Q340A, F346A, F347A]-3XFLAG)*	This Study
yMBS023	*ade2-1 trp1-1 leu2-3,112 his3-11,15 ura3-1 can1-100 bar1::HisG lys2::HisG pep4Δ::unmarked his3::pYMBS001 (GAL1,10-RFC2, RFC3) ura3::pYMBS002 (GAL1,10-RFC4, RFC5) leu2::pYMBS030 (GAL1,10-ELG1[ΔC536]-3XFLAG)*	This Study
yMBS024	*ade2-1 trp1-1 leu2-3,112 his3-11,15 ura3-1 can1-100 bar1::HisG lys2::HisG pep4Δ::unmarked his3::pYMBS001 (GAL1,10-RFC2, RFC3) ura3::pYMBS002 (GAL1,10-RFC4, RFC5) leu2::pYMBS031 (GAL1,10-ELG1[ΔC684]-3XFLAG)*	This Study
yMBS025	*ade2-1 trp1-1 leu2-3,112 his3-11,15 ura3-1 can1-100 bar1::HisG lys2::HisG pep4Δ::unmarked his3::pYMBS001 (GAL1,10-RFC2, RFC3) ura3::pYMBS002 (GAL1,10-RFC4, RFC5) leu2::pYMBS032 (GAL1,10-ELG1[ΔN215]-3XFLAG)*	This Study
yMBS1003	*ade2-1 trp1-1 leu2-3,112 his3-11,15 ura3-1 can1-100 bar1::HisG lys2::HisG pep4Δ::unmarked his3::pYMBS1001 (GAL1,10-hRFC2, hRFC3) ura3::pYMBS1002 (GAL1,10-hRFC4, hRFC5) leu2::pYMBS1003 (GAL1,10-hRFC1-3XFLAG)*	This Study

yMBS1004	*ade2-1 trp1-1 leu2-3,112 his3-11,15 ura3-1 can1-100 bar1::HisG lys2::HisG pep4Δ::unmarked his3::pYMBS1001 (GAL1,10-hRFC2, hRFC3) ura3::pYMBS1002 (GAL1,10-hRFC4, hRFC5) leu2::pYMBS1003 (GAL1,10-ATAD5 [ΔN692]-3XFLAG)*	This Study
yMBS1005	*ade2-1 trp1-1 leu2-3,112 his3-11,15 ura3-1 can1-100 bar1::HisG lys2::HisG pep4Δ::unmarked his3::pYMBS1001 (GAL1,10-hRFC2, hRFC3) ura3::pYMBS1002 (GAL1,10-hRFC4, hRFC5) leu2::pYMBS1003 (GAL1,10-ATAD5-3XFLAG)*	This Study
yMBS1006	*ade2-1 trp1-1 leu2-3,112 his3-11,15 ura3-1 can1-100 bar1::HisG lys2::HisG pep4Δ::unmarked his3::pYMBS1001 (GAL1,10-hRFC2, hRFC3) ura3::pYMBS1002 (GAL1,10-hRFC4, hRFC5) leu2::pYMBS1003 (GAL1,10-ATAD5 [693-1719]-3XFLAG)*	This Study
yMBS1007	*ade2-1 trp1-1 leu2-3,112 his3-11,15 ura3-1 can1-100 bar1::HisG lys2::HisG pep4Δ::unmarked leu2::pYMBS1003 (GAL1,10-ATAD5 [ΔN692]-3XFLAG)*	This Study
yMBS1007	*ade2-1 trp1-1 leu2-3,112 his3-11,15 ura3-1 can1-100 bar1::HisG lys2::HisG pep4Δ::unmarked leu2::pYMBS1003 (GAL1,10-ATAD5 [693-1719]-3XFLAG)*	This Study

Supplementary Table 2. Plasmids used in this study to express ATAD5 in human cells.

Plasmids	Description	Source/ Reference
pMBS1	pcDNA3.1 (ATAD5 1-1844)	Lee et al., 2010 [1]
pMBS2	pcDNA3.1 (ATAD5 1-32+158-1844), ΔN157	This study
pMBS3	pcDNA3.1 (ATAD5 1-32+693-1844), ΔN692	This study
pMBS4	pcDNA3.1 (ATAD5 1-32+984-1844), ΔN983	This study
pMBS5	pcDNA3.1 (ATAD5 1-983), ΔC984	This study
pMBS6	pcDNA3.1 (ATAD5 1-1411), ΔC1412	This study
pMBS7	pcDNA3.1 (ATAD5 1-1601), ΔC1602	This study
pMBS8	pcDNA3.1 (ATAD5 1-32+1602-1844), 1602-1844	This study
pMBS9	pcDNA3.1 (ATAD5 1-32+1720-1844), 1720-1844	This study
pMBS10	pcDNA3.1 (ATAD5 1-32+693-1719), 693-1719	This study
pMBS11	pcDNA5/FRT/TO (ATAD5 1-1844), CM1	This study
pMBS12	pcDNA5/FRT/TO (ATAD5 1-1844), CM2	This study
pMBS13	pcDNA5/FRT/TO (ATAD5 1-1844), CM3	This study
pMBS14	pcDNA5/FRT/TO (ATAD5 1-1844), CM4	This study
pMBS15	pcDNA5/FRT/TO (ATAD5 1-32+401-1844), ΔN400	This study
pMBS16	pMBS15 K1138A, ΔN400 KA	This study
pMBS17	pMBS15 E1305NE1306QD1308A, ΔN400 NQA	This study
pMBS18	pMBS15 E1173K, ΔN400 EK	This study
pMBS19	pcDNA5/FRT/TO (ATAD5 1-32+401-1719), ΔN400 ΔC1720	This study
pMBS20	pcDNA5/FRT/TO (RFC1 1-1147)	This study
pMBS21	pcDNA5/FRT/TO (RFC1 1-834)	This study
pMBS22	pcDNA5/FRT/TO (RFC1 835-1147)	This study
pMBS23	(ATAD5 1-32+693-1598)+(ATAD5 1601-1844), ATAD5 ΔN692 CA	This study
pMBS24	(ATAD5 1-32+693-1598)+(RFC1 835-1147), ATAD5 ΔN692 CR	This study
pMBS25	pcDNA3.1 (ATAD5 1-693)	This study

Supplementary Methods

Preparation of 1.4 Kbps DNA

Sequence:

GATCGGTGCGGGCCTCTTCGCTATTACGCCAGCTGGCGAAAGGGGGATGTGCTGCAAGGCGATT
AAGTTGGGTAACGCCAGGGTTTTCCCAGTCACGACGTTGTAAAACGACGGCCAGTGAATTCCACA
TGTTAAAATAGTGAAGGAGCATGTTCGGCACACAGTGGACCGAACGTGGGGTAAGTGCACTAGG
GTCCGGTTAAACGGATCTCGCATTGATGAGGCAACGCTAATTATCAACATATAGATTGTTATCTATCT
GCATGAACACGAAATCTTTACTTGACGACTTGAGGCTGATGGTGTTTATGCAAAGAAACCACTGTG
TTTAATATGTGTCACTGTTTGATATTACTGTCAGCGTAGAAGATAATAGTAAAAGCGGTTAATAAGTG
TATTTGAGATAAGTGTGATAAAGTTTTTACAGCGAAAAGACGATAAATACAAGAAAATGATTACGAG
GATACGGAGAGAGGTATGTACATGTGTATTTATATACTAAGCTGCCGGCGGTTGTTTGCAAGACCG
AGAAAAGGCTAGCAAGAATCGGGTCATTGTAGCGTATGCGCCTGTGAACATTCTCTTCAACAAGTT
TGATTCCATTGCGGTGAAATGGTAAAAGTCAACCCCCTGCGATGTATATTTTCCTGTACAATCAATC
AAAAAGCCAAATGATTTAGCATTATCTTTACATCTTGTTATTTTACAGATTTTATGTTTAGATCTTTTAT
GCTTGCTTTTCAAAAGGCCTGCAGGCAAGTGCACAAACAATACTTAAATAAATACTACTCAGTAATA
ACCTATTTCTTAGCATTTTTGACGAAATTTGCTATTTTGTTAGAGTCTTTTACACCATTTGTCTCCAC
ACCTCCGCTTACATCAACACCAATAACGCCATTTAATCTAAGCGCATCACCAACATTTTCTGGCGTC
AGTCCACCAGCTAACATAAAATGTAAGCTTTCGGGGCTCTCTTGCCTTCCAACCCAGTCAGAAATC
GAGTTCCAATCCAAAAGTTCACCTGTCCCACCTGCTTCTGAATCAAACAAGGGAATAAACGAATGA
GGTTTCTGTGAAGCTGCACTGAGTAGTATGTTGCAGTCTTTTGGAAATACGAGTCTTTTAATAACTG
GCAAACCGAGGAACTCTTGGTATTCTTGCCACGACTCATCTCCATGCAGTTGGACGATATCAATGC
CGTAATCATTGACCAGAGCCAAAACATCCGCATGCAAGCTTGGCGTAATCATGGTCATAGCTGTTT
CCTGTGTGAAATTGTTATCCGCTCACAATTCCACACAACATACGAGCCGGAAGCATAAAGTGTAAA
GCCTGGGGTGCCTAATGAGTGAGCTAACTCACATTAATTGCGTTGCGCTCACTGCCCGCTTTCC

Amplifying oligonucleotides:

5’-Biotin-GATCGGTGCGGGCCTCTTCGC

GGAAAGCGGGCAGTGAGCG

Annealed oligonucleotides:

CGAAGAGGCCCGCACCGATC

CGTTGCCTCATCAATGCGAGATCC

CCGCTTTTACTATTATCTTCTACGCTGACAG

CACAGGCGCATACGCTACAATGAC

GCACTTGCCTGCAGGCCTTTTG

GCTGGTGGACTGACGCCAG

CTACTCAGTGCAGCTTCACAGAAACCTC

CGCCAAGCTTGCATGCGGATG

GGAAAGCGGGCAGTGAGCG

DNA strands for single molecule experiments

Strand 1

DNA1: 5'-CCC AGT TGA GCG CTT GCT AGG GTT TTT TTT TTT TTT TTT TTT AGA GCA GAT TGT ACC GAG AGT GCA CCA TAT GC /3AmMC6T/-3'

DNA2: 5'-CCC AGT TGA GCG CTT GCT AGG GTT TTT TTT TTT TTT TTT TT /iAmMC6T/ A GAG CAG ATT GTA CCG AGA GTG CAC CAT ATG C-3'

DNA3: 5'-/5DigN/ CCC AGT TGA GCG CTT GCT AGG GTT TTT TTT TTT TTT TTT TTT AGA GCA GAT TGT ACC GAG AGT GCA CCA TAT GC-3'

Strand 2

5'-/5Biosg/ GCA TAT GGT GCA CTC TCG GTA CAA TCT GCT CT-3'

Strand 3

DNA1/DNA2: 5'-CCC TAG CAA GCG CTC AAC TGG G /3DigN/-3'

DNA3: 5'-/5AmMC6/ CCC TAG CAA GCG CTC AAC TGG G-3’

Primers for DNA constructs

pFB-His-PCNA-Flag-F: GAGACGGATCCATGTTCGAGGCGCG

pFB-His-PCNA-Flag-R: GTCTCGCATGCAGATCCTTCTTCATCCTCG

hATAD5FXhoI: GAGACCTCGAGGATGGTGGGGGTCCTGGCC

hATAD5RSphI: GAGACGCATGCAGGGAAGTCAGCTGCCAAAG

hATAD5NLSNheIR: GAGACGGATCCGCCTCCGCTAGCGTCATCATCTTTCTTTCGCTTTTTGC

hATAD5dN157NheI: GAGACGCTAGCGTTTTACGTTACAAGAAACAAGTAGAGGTAC

hATAD5dN400NheI: GAGACGCTAGCGAAGAAAGACAGCAATTTATGA AAGCATTTAGG

hATAD5dN692NheI: GAGACGCTAGCGCAAGCAATACTTCAAAAAACATATCAAAAGC

hATAD5dN983NheI: GAGACGCTAGCAAACAAGAACTGGAGGCTGATGTC

hATAD5dN1602NheI: GAGACGCTAGCGAAGAAAGCAAAACCGGAGACG

hATAD5dN1720NheI: GAGACGCTAGCTGTTCTTCTGCTATTTCAAAAGCATTG

hATAD5dC984BamHIR:
GAGACGGATCCCTAAGGCGCGCCACTATGACACTCGGAAGAAAGTACTTG

hATAD5dC1412BamHIR:
GAGACGGATCCCTAAGGCGCGCCTAAAACTCCACCTCCACTTCTAATCC

hATAD5dC1602BamHIR:
GAGACGGATCCCTAAGGCGCGCCTGCATTTGAGGAAGATGATACAGAGG

hATAD5dC1719BamHIR: GAGACGGATCCTTATTTAGTA AAGCTGAGAGCTTCTGCAGC

hRFC1FXhoI: GAGACCTCGAGGATGGACATTCGGAAATTCTTTGGAG

hRFC1RSphI: GAGACGCATGCTTTCTTCGAACTTTTTCCTTTTCCTTTTCTG

hRFC1dN554: GAGACCTCGAGGATGAAGGAGCAGGTGGCTGAGG

hRFC1c: GAGACGCATGCTTTCTTCGAACTTTTTCCTTTTCC

XhoI-RAD17: GAGACCTCGAGGATGAATCAGGTAACAGACTGGG

RAD17-SphI: GTCTCGCATGCTGTCCCATCACTCTCGTAGTCTTC

pFB-His-CTF18-SII-Flag-F: GAGACGGATCCATGGAGGACTACGAGCAG

pFB-His-CTF18-SII-Flag-R: GTCTCGCGGCCGCCTACTTGTCATCGTCATCCT TG

pFL-RAD6B-F: GAGACCCCGGGATGTCGACCCCGGC

pFL-RAD6B-R: GTCTCGCTAGCTTATGAATCATTCCAGCTTTGTTCAAC

pFB-His-RAD18-Flag-F: GAGACCTCGAGGATGGACTCCCTGGCC

pFB-His-RAD18-Flag-R: GTCTCGCATGCTTAATTCCTATTACGCTTGTTTCTTGGTTC

pFL-hUBC13-hMMS2-oJP10: CTCCCGGTACCGCATCAAATATTATTCATGGC

pFL-hUBC13-hMMS2-oJP3:
GAAAACCTGTATTTTCAGGGCGCCATGGATGCCGGGCTGCCCCGCAGGAT

pFL-hUBC13-hMMS2-oJP8:
CGGAATTCAAAGGCCTACGTCGACGAGCTCATGGCGGTCTCCACAGGAGT

pFL-hUBC13-hMMS2oJP6:
ATCACCGTCATGGTCTTTGTAGTCGCATGCATTGTTGTATGTTTGTCCTT

HLTF-5': GATCCTCGAGGATGTCCTGGATGTTCAAGAGGG

HLTF-3': GTAGTCGCATGCTAAGTCAATTAATGTTCTGATTTC

RFC1-dC834-AscI: GTCTCGGCGCGCCAGCTTTGGCCTGGTCATAG

AflII-RFC1: GAGACCTTAAGATGGACATTCGGAAATTCTTTG

RFC1-AscI: GTCTCGGCGCGCCTTTCTTCGAACTTTTTCC

AflII-RFC1-dN835: GAGACCTTAAGGATTCTCACAGAGCCAAAAA

ATAD5-693-BamKpn:
GAGACGGATCCGGTACCATGGCAAGCAATACTTCAAAAAACATATCAAAAGC

ATAD5-1598-R-NheSph : GAGACGCATGCGCTAGCGGAAGATGATACAGAGGACAGGC

ATAD5-1600-BamKpnNhe:
GAGACGGATCCGGTACCGCTAGCGCAGAAGAAAGCAAAACCGGAGAC

ATAD5-Sph : GAGACGCATGCAGGGAAGTCAGCTGCCAAAGTATTCAC

RFC1-555-XhoKpn: GAGACCTCGAGGGTACCATGCAGGTGGCTGAGGAGACAAGTGGTGAC

RFC1-833-NheSph: GAGACGCATGCGCTAGCGGCCTGGTCATAGGTTAATGC

RFC1-835-XhoKpnNhe: GAGACCTCGAGGGTACCGCTAGCGATTCTCACAGAGCCAAAAAGG

RFC1-Sph: GAGACGCATGCTTTCTTCGAACTTTTTCCTTTTCC

ATAD5RLCMT1 :
GCTCTGGAGAATTAAAGGCAGCTGCAGCAGCTGGCGGCGGTACTAAATGTTCTTCTGCTATTTCA
AAAG

ATAD5RLCMT2:
GCTTTACTAAATGTTCTTCTGCTATTGCAGCAGGAGGGGCAACCTTGAATTCTTGCAAGAAATTA
GG

ATAD5RLCMT3:
CCCTTCGAAACATCTGTAAGACTGCGGCGGGAGCAGCACAAGGAAAAAGTAAAAGAAGATTCC

ATAD5RLCMT4:
GAAGCTAAAAGAACAAGGAAAAAGTGCAGCAGCAGGCGGGCACTATTTTGAAGGAATTCATCTT
G

ATAD51305EENQT: GCAACATCTCTTATTCTTTTTAACCAGGTTGATGTAATTTTTGATGAA

ATAD5EED1308AT: CTTATTCTTTTTAACCAGGTTGCTGTAATTTTTGATGAAGATGCTGGG

ATAD5E1173KT: CAAATTCTATCTCAGTTGAAGAAAGCTACTCAGTCCCATCAA

BamHI-hRFC2-F: GAGACGGATCCATGGAGGTGGAGGCCG

XhoI-hRFC2-R: GAGACCTCGAGCTAACTGGCCACCGGGG

BamHI-hRFC3-F: GAGACGGATCCATGAGCCTCTGGGTGGAC

XhoI-hRFC3-R: GAGACCTCGAGTCAGAACATCATGCCTTCCAATCC

BamHI-hRFC4-F: GAGACGGATCCATGCAAGCATTTCTTAAAGGTACATCC

XhoI-hRFC4-R: GAGACCTCGAGTTAACAATTCTGAGATAACTGCTGCATC

BamHI-hRFC5-F: GAGACGGATCCATGGAGACCTCAGCACTCAAG

XhoI-hRFC5-R: GAGACCTCGAGCTAGGCCTCTGCAACAATCAG

Supplementary Reference

1 Lee, K. Y. *et al.* Human ELG1 regulates the level of ubiquitinated proliferating cell nuclear antigen (PCNA) through Its interactions with PCNA and USP1. *J Biol Chem* **285**, 10362-10369, doi:10.1074/jbc.M109.092544 (2010).
